# The use of AI tools in academic writing and its associations with college students' critical thinking and academic integrity: a path analysis involving self-regulated learning and innovative behavior

**DOI:** 10.3389/fpsyg.2026.1845571

**Published:** 2026-07-08

**Authors:** Xiaofei Zhen, Xiaolian Lian

**Affiliations:** 1 School of Translation and Interpreting Studies, Heilongjiang University, Harbin, Heilongjiang, China; 2 School of Foreign Languages and Literature, Heilongjiang University, Harbin, Heilongjiang, China

**Keywords:** academic integrity, AI tool use, critical thinking, innovative behavior, self-regulated learning

## Abstract

**Background:**

AI tools are increasingly used in college students' academic writing, raising concerns about critical thinking and academic integrity. Prior work has focused mainly on writing performance, whereas psychological and behavioral pathways remain less examined. Guided by Social Cognitive Theory, this study examined a path model linking AI tool use with critical thinking and academic integrity via self-regulated learning and innovative behavior.

**Methods:**

A cross-sectional survey was conducted with 946 undergraduates from five universities in China. Measures included AI tool use, self-regulated learning, innovative behavior, critical thinking, and academic integrity. The hypothesized model was tested using PLS-SEM with bootstrapping.

**Results:**

AI tool use was positively associated with self-regulated learning and innovative behavior. Both intermediaries were positively associated with critical thinking and academic integrity. Bootstrapping supported significant indirect associations from AI tool use to both outcomes through self-regulated learning and innovative behavior.

**Conclusion:**

AI tool use in academic writing was associated with higher levels of critical thinking and academic integrity, with self-regulated learning and innovative behavior representing intermediary variables in these associations. The findings may inform AI-supported writing instruction and integrity guidance in higher education.

## 1 Introduction

The rapid uptake of artificial intelligence (AI) tools, including text generation, grammar checking, and literature review assistants, is reshaping college students' academic writing. These tools may be associated with greater efficiency and fewer technical barriers, yet they also intensify debates about critical thinking and academic integrity (Jankovic and Kulic, 2025). Critical thinking involves reflective judgement, questioning assumptions, and analytical reasoning (Scriven and Paul, 1987). Academic integrity refers to compliance with ethical standards in academic work (Macfarlane et al., 2014). Understanding how AI supported writing is associated with these competencies has therefore become an important issue in higher education.

Existing evidence points to mixed patterns. AI writing support may coincide with students' efforts to address language constraints, revise their writing, and consider alternative arguments, which may be associated with stronger critical engagement (Hooda et al., 2022). At the same time, heavy reliance on AI generated content has been linked to concerns about reduced deep processing and weaker independent evaluation (Hosseini et al., 2023). Similar tensions appear in research on academic integrity. AI tools have also been associated with norm related practices such as standardized referencing (Hysaj et al., 2024), while the opacity of content generation raises concerns about attribution and misconduct risks (Kong et al., 2024). Despite this growing literature, fewer studies have examined the association patterns and indirect links connecting AI tool use with critical thinking and academic integrity via psychological and behavioral processes.

To fill this void, this research employs Social Cognitive Theory (SCT) and outlines a three-way interaction model connecting environmental factors, self-regulation, and active participation (Bandura, 2001). AI tools are conceptualized as a key feature of the writing environment, while self-regulated learning and innovative behavior are examined as intermediate processes associated with critical thinking and academic integrity. By testing this pathway model among undergraduates, the study aims to clarify how students' regulation and engagement co-occur with key academic competencies in AI supported writing contexts rather than to establish directional or causal effects, and to inform higher education policy and instructional design.

## 2 Theoretical framework and literature review

### 2.1 Theoretical foundation

SCT conceptualizes learning and action as reciprocal relations among personal processes, behavior, and environmental conditions (Bandura, 2001). A central assumption is that individuals exercise agency through self-regulatory processes within specific contexts. Earlier studies utilized SCT to examine competence related learning and ethical conduct, for instance connections between self-perceived ability and skill acquisition (Zhao et al., 2024), as well as how moral disengagement shapes norm related behavior (Helzer et al., 2024). In AI supported academic writing, SCT offers a useful framework for examining how a changing writing environment co-occurs with students' regulation and engagement.

In this study, AI tools are treated as a salient environmental feature of academic writing, given their functions such as automated text generation and real time feedback. Depending on learners‘ use of these tools, such features could relate to either beneficial or challenging writing behaviors. At the individual level, self-regulated learning involves establishing goals, tracking progress, and engaging in reflection when carrying out writing assignments (Zimmerman, 2002). At the behavioral level, innovative behavior reflects students' active exploration and implementation of new ideas in writing. Together, these processes provide a structured way to examine how AI tool use is linked to two key academic competencies, namely critical thinking and academic integrity, within a single pathway model. This SCT based framing supports an integrated analysis of environmental conditions, self-regulation, and behavioral engagement in AI supported writing contexts.

### 2.2 Literature review

The growing use of AI tools in academic writing has prompted renewed discussion about students' critical thinking. A strand of research suggests that AI supported drafting, feedback, and revision can sit alongside activities such as checking coherence, clarifying claims, and refining arguments, which are commonly treated as indicators of critical engagement in writing (Gonsalves, 2024; Poce, 2024; Ruiz-Rojas et al., 2024). AI assisted literature searching and source organization may also be linked to more time available for reading, comparison, and synthesis rather than routine tasks (Hooda et al., 2022). From the perspective of SCT, such tools can be understood as a feature of the learning environment that co-occurs with students' engagement and self-regulation in writing tasks (Bandura, 2001). At the same time, a second strand of research raises concerns about how students work with AI generated outputs. Where reliance becomes routine and verification practices are limited, the use of AI may align with less independent evaluation, weaker reflective judgement, and narrower engagement with evidence (Hosseini et al., 2023). Relying too heavily on AI-produced outputs has been associated with less engagement in deep analysis and with weaker independent cognitive reasoning (McAlister et al., 2024; Van Slyke et al., 2023). AI-generated content's unclear design can hide its reasoning, especially when assessing bias or checking sources (Bearman and Ajjawi, 2023; Hosseini et al., 2023). Furthermore, the opacity of AI generated content can complicate source checking and bias scrutiny, which are central to critical thinking in academic contexts. These mixed patterns suggest that the association between AI tool use and critical thinking may vary across contexts and forms of use, particularly whether AI is approached as support for drafting and evaluation rather than as a substitute for students' own reasoning (Qadir, 2023). However, some studies have reported null or conditional effects, with observed patterns varying by task complexity and students' prior skills (Essien et al., 2024). This mixed evidence warrants further investigation.

Using generative AI for academic writing has raised worries about scholarly honesty, especially regarding who is credited as author, proper citation, and what counts as permitted help. A body of work links AI supported writing to higher risks of misconduct when AI generated text is used without appropriate acknowledgment or critical checking (Bittle and El-Gayar, 2025; Cotton et al., 2024). Related discussions highlight that the accessibility and fluency of AI outputs may encourage over reliance, which can blur boundaries between support and substitution in students' writing practices (Currie, 2023; Kong et al., 2024). From a SCT perspective, integrity related conduct in such contexts is closely connected with learners' self-regulation and the norms operating within the learning environment, including monitoring, judgement, and boundary setting (Bandura, 2001). Meanwhile, research has observed that employing AI does not consistently correspond to reduced ethical standards. Under explicit guidance and clear expectations, AI tools may be used in ways that support integrity related practices, such as improving transparency in the writing process and reinforcing citation awareness (Hysaj et al., 2024; Lund et al., 2025). This mixed evidence suggests that the association between AI tool use and academic integrity may vary across contexts and forms of use.

Self regulated learning refers to learners' active management of cognition, motivation, and behavior in pursuit of academic goals, typically involving planning, monitoring, strategy use, and reflection (Pintrich, 2000; Zimmerman, 2002). In AI supported writing contexts, a growing literature suggests that students' use of AI tools is associated with how they plan and monitor writing tasks and how they respond to feedback and revise their work (Dahri et al., 2024; Ng et al., 2024). SCT perspective, AI tools can be treated as a feature of the learning environment that co occurs with learners' regulation practices during writing (Bandura, 2001). Self-regulated learning is also widely discussed in relation to critical thinking. Students who set goals, monitor progress, and engage in reflection may be more likely to scrutinize claims, evaluate evidence, and revise arguments during writing, which aligns with common accounts of critical thinking in academic work (Akcaoglu et al., 2023; Jin et al., 2025). Furthermore, self-regulated learning connects to academic honesty. When students check their work against guidelines and think about how they cite sources, they are more likely to follow academic rules (Lodge et al., 2023; Simon and Pavlin-Bernardic, 2024).

Innovative behavior refers to activities such as generating new ideas, seeking support for them, and applying them in practice (Sudibjo and Prameswari, 2021). In academic writing, it is often reflected in students' willingness to explore alternative perspectives, develop original arguments, and move beyond conventional structures. Recent findings indicate that when students use AI tools for learning and writing, they tend to show greater creative involvement and more innovative inclinations (Ji et al., 2025; Ma et al., 2024). From a SCT perspective, innovative behavior can be viewed as a form of active engagement through which learners work with environmental resources, including AI tools, while organizing and refining their work (Bandura, 2001). Innovative behavior has also been discussed in relation to critical thinking. When students generate and test ideas, compare options, and justify novel claims, these practices align with evaluation and reasoning processes typically associated with critical thinking (El-Sayed et al., 2025; Li et al., 2024). According to SCT, learners who employ AI tools to examine different viewpoints, produce original concepts, and try out various writing formats are actively reshaping environmental information (Bandura, 2001). This active engagement has been associated with a stronger sense of ownership over their written work, as students invest effort in refining and personalizing AI-generated suggestions rather than passively accepting them. A heightened sense of ownership, in turn, has been associated with greater attention to proper attribution and reduced likelihood of academic misconduct (Eshet and Margaliot, 2022). Thus, innovative behavior can be understood as an accompanying behavioral process that co-occurs with students' internalization of and responsibility for AI-assisted writing, rather than merely as a proxy for creativity. In addition, students who engage in innovation-oriented practices may also monitor their work more carefully, checking whether sources are properly credited and whether AI-generated content has been sufficiently transformed to reflect their own voice (Krou et al., 2021).

**Figure 1:**
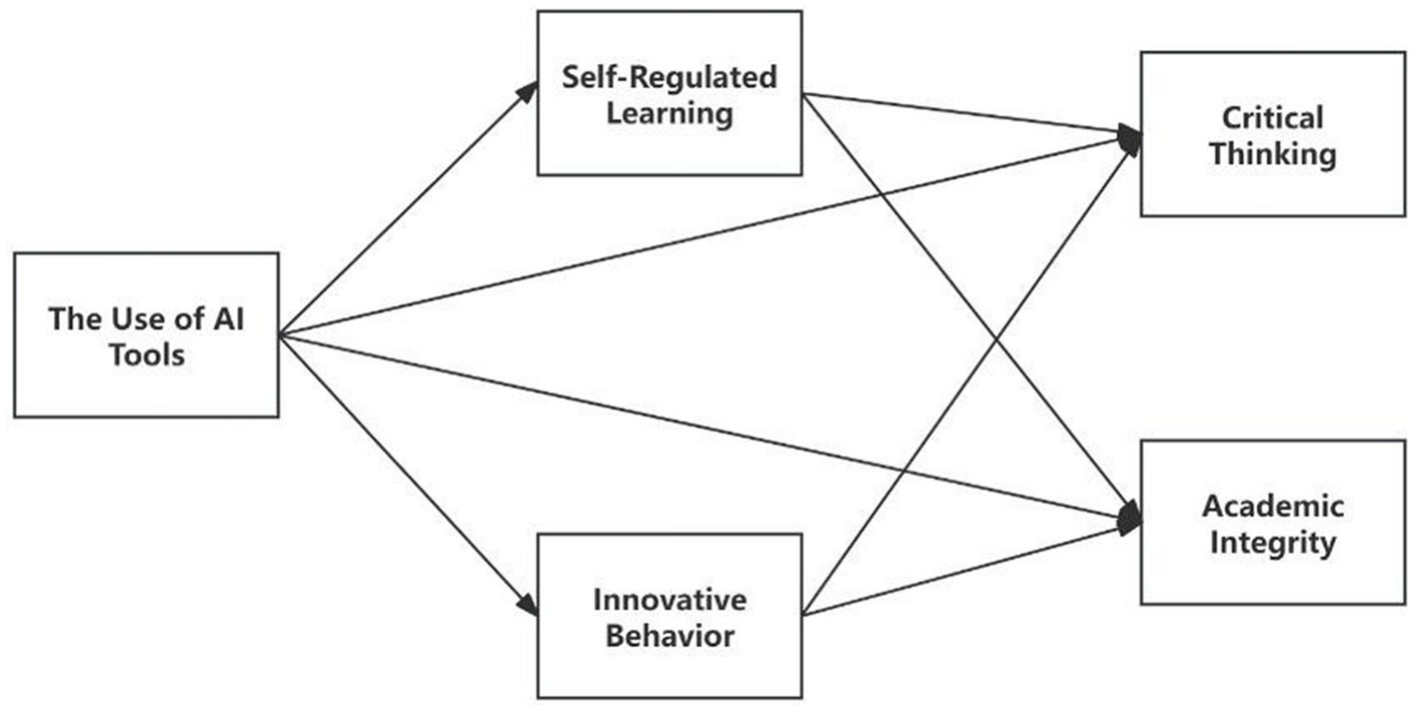
Hypothesized research model.

By integrating self-regulated learning and innovative behavior into the Social Cognitive Theory framework, this study enhances our understanding of how AI tool use in academic writing relates to college students' critical thinking and academic integrity. It extends SCT by showing how environmental conditions (AI tools) are associated with these psychological and behavioral processes. This study clarifies how using AI tools relates to cognitive and ethical skills. It also offers a framework that may inform strategies intended to encourage responsible AI use in higher education. The following hypotheses are put forth in this study based on this framework, and the conceptual model is presented in Figure 1:

**H1:** The use of AI tools in academic writing is positively associated with critical thinking.

**H2:** The use of AI tools in academic writing is negatively associated with academic integrity.

**H3:** Self-regulated learning shows an indirect association in the relationship between AI tool use and critical thinking.

**H4:** Self-regulated learning shows an indirect association in the relationship between AI tool use and academic integrity.

**H5:** Innovative behavior shows an indirect association in the relationship between AI tool use and critical thinking.

**H6:** Innovative behavior shows an indirect association in the relationship between AI tool use and academic integrity.

## 3 Method

### 3.1 Sample and data collection

The research received approval from Heilongjiang University's Ethics Review Committee and took place at five universities within Heilongjiang Province, China. All procedures adhered to the ethical standards outlined in the Declaration of Helsinki. An online survey was administered using Wenjuanxing, a professional survey platform, to collect data from February to March 2026. After obtaining institutional approval, class rosters were obtained from each university's academic affairs office. Students were then randomly selected from these rosters using computer-generated numbers, and faculty members disseminated the survey link. The survey invitation emphasized voluntary participation and strict privacy protection. Written consent was willingly submitted by each individual before they took part in the research. Throughout the study, we ensured that no sensitive or traceable personal data were recorded or reported.

Following Kline (2018) recommendation of a minimum of 10 respondents per item, we estimated the required sample size for our 43-item questionnaire as 516, after adjusting for a projected 20% exclusion rate. To ensure robust data collection, we distributed 1,000 questionnaires and successfully collected all 1,000 responses. During the quality screening process, we removed 54 cases due to excessive missing values (over 20%) or a high frequency of extreme responses (over 80% marked “strongly agree” or “strongly disagree”), which are known to produce biased results through floor or ceiling effects (Wang et al., 2012). Ultimately, we retained 946 valid questionnaires for analysis, yielding a completion rate of 94.6%.

Table 1 summarizes the background characteristics of the final sample, including major demographic attributes. Among the participants, 451 (47.70%) were male and 495 (52.30%) were female. Of all surveyed individuals, 71.80% (*n* = 680) were aged between 18 and 22 years, representing the dominant age segment in the sample. Most were third-year students (39.30%, *n* = 372), and a significant proportion majored in humanities and social sciences (39.00%, *n* = 369). In addition, 60.90 percent (n = 576) of participants reported frequent use of AI tools.

**Table 1:** Demographic characteristics of the participants.

Variable	Category	Frequency (*n*)	Percentage (%)
Gender	Male	451	47.70
	Female	495	52.30
Age	18 and below	136	14.40
	18–22	680	71.80
	Above 22	130	13.70
Year of study	First year	233	24.60
	Second year	195	20.60
	Third year	372	39.30
	Fourth year	146	15.40
Field of study	Science and Engineering	47	5.00
	Economics and Management	247	26.10
	Humanities and Social Sciences	369	39.00
	Arts	218	23.00
	Others	65	6.90
Frequency of AI tool use	Rarely	12	1.30
	Occasionally	318	33.60
	Frequently	576	60.90
	Very frequently	40	4.20

### 3.2 Measurement instruments

The questionnaire comprised two main components. The initial section focused on gathering background characteristics of participants, while the subsequent section explored the core theoretical constructs under investigation. Except for demographic questions, all items were scored on a five-point scale from 1 (strongly disagree) to 5 (strongly agree). Table 2 summarizes five constructs with item counts from 3 to 17 and Cronbach's α values between 0.859 and 0.956, all taken or adjusted from existing validated instruments.

**Table 2:** Summary of Measurement Instruments.

Construct	Source	Number of Items	Cronbach's α
The Use of AI Tools	Man Tang et al. (2022)	3	0.899
Self-regulated learning	Zimmerman (2000)	4	0.942
Innovative behavior	Ng and Lucianetti (2016)	9	0.956
Critical thinking	Zhou et al. (2024)	5	0.859
Academic integrity	Ramdani (2018)	17	0.872

#### 3.2.1 The use of AI tools

This research employed a three-item measure originally created by Man Tang et al. (2022) for organizational contexts to assess AI tool usage. To establish content validity for the academic writing context, we adapted the scale by rewording the items to specifically reference academic writing (e.g., “I spent most of my academic writing time working with artificial intelligence.”). In this study, “AI tools” were operationally defined as software or applications that assist with academic writing tasks, including generative AI (e.g., ChatGPT for text generation), assistive AI (e.g., Grammarly for grammar checking), and organizational AI (e.g., Connected Papers for literature mapping). We also provided a clear definition of “AI tools” for those taking part in the study. These were described as any software or applications that help with academic writing, such as large language models (e.g., ChatGPT), grammar checkers (e.g., Grammarly), and literature mapping tools (e.g., Connected Papers), though not limited to these examples. Empirical evidence from Chinese student cohorts has demonstrated the instrument's measurement consistency and construct adequacy (Zhang et al., 2025).

#### 3.2.2 Self-regulated learning

Self-regulated learning was assessed using four items adapted from Zimmerman (2000) original scale. One example statement reads: “When employing AI tools for scholarly writing, I pose questions to myself that help me concentrate on reading and comprehending the content.” The Chinese version has demonstrated strong psychometric properties in previous studies (Zhou et al., 2024).

#### 3.2.3 Innovative behavior

To measure innovative behavior, this research applied a nine-item scale from Ng and Lucianetti (2016). An example item is: “I generate original ideas for making things better.” The scale has been empirically tested and shown to be reliable within samples of Chinese college students (Ma et al., 2024).

#### 3.2.4 Critical thinking

To assess critical thinking, five items were drawn from the Motivated Strategies for Learning Questionnaire—an instrument initially designed by Pintrich (1991) and later modified by Zhou et al. (2024) to suit contemporary academic contexts. An example item is: “I often question information obtained from AI tools to evaluate its persuasiveness.” Research carried out inside China's school system has verified that this measure is both reliable and valid (Zhou et al., 2024). In this study, confirmatory factor analysis yielded factor loadings ranging from 0.772 to 0.816, supporting construct validity without indicating item redundancy.

#### 3.2.5 Academic integrity

To evaluate academic integrity, this study used a 17-item measure first created by Ramdani (2018) and later modified by Bin-Nashwan et al. (2023). A sample item is: “To me, academic integrity begins with self-reflection and authentic expression.” This scale measures students' attitudes toward academic integrity rather than their actual integrity behaviors (e.g., frequency of plagiarism). We acknowledge this as a conceptual limitation; thus, our findings reflect attitudinal orientations rather than behavioral frequencies. The Chinese version demonstrated robust psychometric properties.

This study employed a rigorous forward-backward translation protocol for all original English instruments. Two bilingual experts—one content specialist and one linguist—first translated the items into Chinese. Afterwards, two separate specialists each carried out an independent back-translation of the items into English. Any differences between the back-translated texts and the original statements, such as shifts in meaning or concept, were settled in group discussions with all four translators. If no consensus was achieved, an additional bilingual specialist in content was brought in to decide the final outcome. The pilot test was then conducted with 20 participants from the target population. The pilot results were evaluated both quantitatively and qualitatively. Quantitatively, we calculated item-level clarity scores (all items averaged above 4.0 on a 5-point scale) and scale-level Cronbach's α (ranging from 0.85 to 0.94). Qualitatively, we collected written and verbal feedback on wording ambiguity, item interpretation, and cultural appropriateness. After the pilot, we slightly reworded some items for clarity, such as splitting double-barreled questions. No items were removed as a result of the pilot test. The final Chinese version was then used in the formal survey.

### 3.3 Data analysis

We used SPSS 26.0 and SmartPLS 4.0 for data analysis. SPSS computed descriptive statistics such as means, standard deviations, skewness, and kurtosis. In SmartPLS 4.0, we evaluated the measurement model by examining outer loadings, Cronbach's alpha, composite reliability (CR), and average variance extracted (AVE). Discriminant validity was assessed with the HTMT and Fornell–Larcker criteria. For the structural model, we applied PLS-SEM with 5,000 bootstrap resamples to estimate path coefficients and significance. Collinearity was checked using variance inflation factor (VIF). Model explanatory power was assessed with R^2^, and predictive relevance with Q^2^. Following Sarstedt et al. (2021), PLS-SEM was adopted due to its suitability for complex models and bootstrapping-based inference.

## 4 Results

### 4.1 Descriptive statistical analysis

We summarized the descriptive statistics for the study variables in Table 3. Following (Kline 2023) distributional normality thresholds (|Sk| < 2, |Kur| < 7), we confirmed all variables met criteria for normal distribution.

**Table 3:** Descriptive statistics.

Variable	Sk	Kur	M±SD
AIU	−0.147	−0.670	2.976 ± 1.023
SRL	−0.208	−0.781	2.874 ± 0.919
IB	−0.765	−0.649	3.238 ± 0.953
CT	−0.252	−0.616	2.912 ± 0.860
AcInt	−0.233	−0.626	2.930 ± 0.816

N = 946. Sk, skewness; Kur, kurtosis; M, mean; SD, standard deviation. AIU, The Use of AI Tools; SRL, Self-Regulated Learning; IB , Innovative Behavior; CT, Critical Thinking; AcInt, Academic Integrity.

### 4.2 Common method bias

We used Harman's single-factor test (Podsakoff et al., 2003) to check whether common method bias posed a threat, as all data were self-reported in one survey. The first factor explained 49.524% of the overall variance, under the suggested 50% cutoff. This indicates that common method bias is unlikely to be a serious concern in this study (Podsakoff et al., 2003).

### 4.3 SEM analysis

#### 4.3.1 Measurement model

We examined the measurement model's reliability and validity based on Leguina (2015) and Hair Jr et al. (2021). Every outer loading was above 0.708, while Cronbach's alpha and CR values pointed to good internal consistency (Table 4). Convergent validity was confirmed as all AVE scores exceeded 0.50. Table 5 shows every HTMT value remained below the suggested 0.85 cutoff (Gefen et al., 2011). Table 6 also shows that each construct's square root of AVE exceeded its correlations with other constructs, thus meeting the Fornell–Larcker criterion (Fornell and Larcker, 1981). These results collectively support satisfactory discriminant validity across all constructs.

**Table 4:** Reliability and validity.

Constructs	Items	Outer loadings	Cronbach' α	CR	AVE
AIU	AIU 1	0.920	0.899	0.937	0.832
	AIU 2	0.905			
	AIU 3	0.911			
SRL	SRL 1	0.893	0.942	0.951	0.683
	SRL2	0.901			
	SRL3	0.801			
	SRL4	0.805			
IB	IB 1	0.840	0.956	0.960	0.586
	IB 2	0.825			
	IB 3	0.845			
	IB 4	0.812			
	IB 5	0.799			
	IB 6	0.751			
	IB 7	0.886			
	IB 8	0.815			
	IB 9	0.857			
CT	CT 1	0.772	0.859	0.899	0.640
	CT 2	0.816			
	CT 3	0.810			
	CT 4	0.804			
	CT 5	0.797			
AcInt	AcInt 1	0.803	0.872	0.913	0.724
	AcInt 2	0.808			
	AcInt 3	0.805			
	AcInt 4	0.718			
	AcInt 5	0.737			
	AcInt 6	0.739			
	AcInt 7	0.763			
	AcInt 8	0.759			
	AcInt 9	0.757			
	AcInt 10	0.762			
	AcInt 11	0.748			
	AcInt 12	0.784			
	AcInt 13	0.760			
	AcInt 14	0.795			
	AcInt 15	0.762			
	AcInt 16	0.753			
	AcInt 17	0.750			

AIU, The Use of AI Tools; SRL, Self-Regulated Learning; IB, Innovative Behavior; CT, Critical Thinking; AcInt, Academic Integrity.

**Table 5:** Discriminant validity (HTMT Criterion).

Construct	HTMT value
CT ↔ AcInt	0.785
IB ↔ AcInt	0.682
IB ↔ CT	0.724
SRL ↔ AcInt	0.793
SRL ↔ CT	0.809
SRL ↔ IB	0.745
AIU ↔ AcInt	0.662
AIU ↔ CT	0.701
AIU ↔ IB	0.819
AIU ↔ SRL	0.726

AIU, The Use of AI Tools; SRL, Self-Regulated Learning; IB, Innovative Behavior; CT, Critical Thinking; AcInt, Academic Integrity.

**Table 6:** Discriminant validity (Fornell-Larcker Criterion).

Construct	AIU	IB	AcInt	CT	SRL
AIU	**0.912**	-	-	-	—
IB	0.754	**0.826**	-	-	—
AcInt	0.613	0.646	**0.765**	-	—
CT	0.617	0.651	0.710	**0.800**	-
SRL	0.642	0.674	0.729	0.700	**0.851**

AIU, The Use of AI Tools; SRL, Self-Regulated Learning; IB, Innovative Behavior; CT, Critical Thinking; AcInt, Academic Integrity. Bold values on the diagonal represent the square root of the average variance extracted (AVE) for each construct. Fornell-Larcker criterion: discriminant validity requires that each construct's AVE square root (diagonal) be greater than its correlations with other constructs.

#### 4.3.2 Structural model

To check for multicollinearity, we analyzed the VIF, using the threshold of 5, which indicates that no severe multicollinearity was present among the predictors (Hair et al., 2011). Table 7 indicates that the VIF values ranged from 1.000 to 2.697, confirming the absence of multicollinearity in this model.

**Table 7:** Collinearity diagnostics (VIF).

Path	VIF
IB → AcInt	2.697
IB → CT	2.697
SRL → AcInt	1.984
SRL → CT	1.984
AIU → AcInt	2.507
AIU → CT	2.507
AIU → IB	1.000
AIU → SRL	1.000

AIU, The Use of AI Tools; SRL, Self-Regulated Learning; IB, Innovative Behavior; CT, Critical Thinking; AcInt, Academic Integrity.

#### 4.3.3 Path hypotheses testing

**Figure 2:**
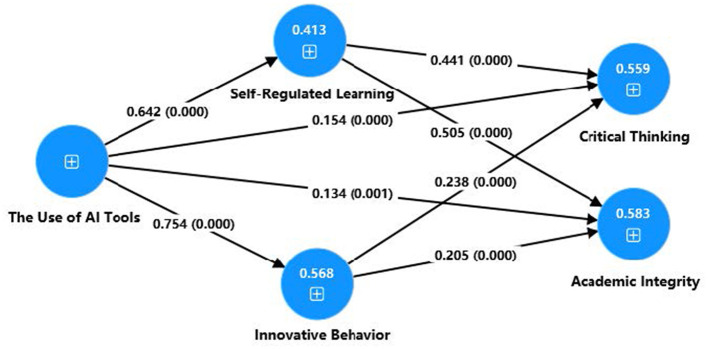
Path coefficients of the structural model.

We tested the hypothesized paths using PLS-SEM combined with 5,000 bootstrap resamples. As shown in Table 8 and Figure 2, the majority of path coefficients reached statistical significance. However, the path from AI tool use to academic integrity (H2), although statistically significant (β = 0.134, *p* = 0.001), was positive rather than negative as hypothesized; therefore, H2 was not supported. AI tool use exhibited markedly varied magnitude of correlational links with different outcome variables. It showed strong associations with innovative behavior (β = 0.754, *p* < 0.001) and self-regulated learning (β = 0.642, *p* < 0.001), and smaller but significant associations with academic integrity (β = 0.134, *p* = 0.001) and critical thinking (β = 0.154, *p* < 0.001). Innovative behavior and self-regulated learning both showed positive links to academic integrity (β = 0.205; β = 0.505) and critical thinking (β = 0.238; β = 0.441). Between the two, self-regulated learning had much larger standardized path coefficients, indicating a stronger correlational relationship.

**Table 8:** Path hypotheses testing.

Hypothesis	Original sample (O)	Sample mean (M)	Standard deviation (STDEV)	*t*	*p*	Results
AIU → IB	0.754	0.754	0.022	34.653	0.000	Supported
AIU → AcInt	0.134	0.134	0.042	3.196	0.001	Not supported
AIU → CT	0.154	0.152	0.035	4.386	0.000	Supported
AIU → SRL	0.642	0.642	0.023	28.498	0.000	Supported
IB → AcInt	0.205	0.205	0.046	4.450	0.000	Supported
IB → CT	0.238	0.240	0.036	6.540	0.000	Supported
SRL → AcInt	0.505	0.505	0.051	9.826	0.000	Supported
SRL → CT	0.441	0.441	0.034	13.041	0.000	Supported

AIU, The Use of AI Tools; SRL, Self-Regulated Learning; IB, Innovative Behavior; CT, Critical Thinking; AcInt, Academic Integrity. O, original sample; M, sample mean; STDEV, standard deviation; t, t-value; p, p-value.

We assessed explanatory power and predictive relevance for the endogenous variables using R^2^ and Q^2^ (Hair Jr et al., 2021). Table 9 shows the model's variance explanation for innovative behavior (*R*^2^ = 0.568), academic integrity (*R*^2^ = 0.583), critical thinking (*R*^2^ = 0.559), and self-regulated learning (*R*^2^ = 0.413), all at moderate levels. All Q^2^ values were above zero, indicating predictive relevance.

**Table 9:** *R*^2^ and *Q*^2^

Construct	R^2^	Q^2^
IB	0.568	0.567
AcInt	0.583	0.374
CT	0.559	0.379
SRL	0.413	0.411

AIU, The Use of AI Tools; SRL, Self-Regulated Learning; IB, Innovative Behavior; CT, Critical Thinking; AcInt, Academic Integrity. R^2^ = explanatory power; Q^2^ = predictive relevance.

Cohen's f^2^ was further calculated to examine the practical effect size of each structural path. Detailed results are presented in Table 10. Notably, the f^2^ value of AI tool use on innovative behavior reaches 1.32, indicating a strong statistical association in this model. This result is theoretically reasonable given that AI application has often been associated with individual innovative thinking and behavioral performance in learning scenarios.

**Table 10:** Path-Specific Effect Sizes (Cohen's *f* ^2^).

Path	Cohen's f^2^	Effect Size
IB → AcInt	0.04	Small
IB → CT	0.05	Small
SRL → AcInt	0.31	Medium
SRL → CT	0.22	Medium
AIU → AcInt	0.02	Small
AIU → CT	0.02	Small
AIU → IB	1.32	Large
AIU → SRL	0.70	Large

AIU, The Use of AI Tools; SRL, Self-Regulated Learning; IB, Innovative Behavior; CT, Critical Thinking; AcInt, Academic Integrity.

### 4.4 Indirect associations

We applied bootstrapping within PLS-SEM to test whether innovative behavior and self-regulated learning accounted for indirect associations in the links from AI tool use to academic integrity and critical thinking. As shown in Table 11, innovative behavior showed a significant indirect effect linking AI tool use with academic integrity (β = 0.155, *t* = 4.439, *p* < 0.001) and critical thinking (β = 0.179, *t* = 6.166, *p* < 0.001), while the corresponding direct paths also remained significant. Similarly, self-regulated learning showed significant indirect effects linking AI tool use with academic integrity (β = 0.324, *t* = 8.798, *p* < 0.001) and critical thinking (β = 0.283, *t* = 11.934, *p* < 0.001), with the direct effects also significant. Further comparison of effect magnitudes shows clear differences across associational paths. Specifically, the indirect association via self-regulated learning was stronger than that via innovative behavior toward both academic integrity (β = 0.324 vs. β = 0.155) and critical thinking (β = 0.283 vs. β = 0.179). In addition, all indirect associational effects were larger than the corresponding direct associations (β = 0.134 and β = 0.154). These results reflect statistically meaningful indirect correlations between AI tool use and the two academic outcome variables. Comparisons of effect magnitudes show that the numerical values of indirect correlations linked to self-regulated learning were relatively higher, and the direct correlational values between AI tool use and academic outcomes still reached notable practical magnitude.

**Table 11:** Indirect associations analysis.

Relationship	Indirect Effect	2.5%	97.5%	*t*	*p*	Direct Effect	*t*	*p*
AIU → IB → AcInt	0.155	0.087	0.225	4.439	0.000	0.134	3.196	0.000
AIU → IB → CT	0.179	0.126	0.240	6.166	0.000	0.154	4.386	0.000
AIU → SRL → AcInt	0.324	0.252	0.394	8.798	0.000	0.134	3.196	0.000
AIU → SRL → CT	0.283	0.237	0.329	11.934	0.000	0.154	4.386	0.000

AIU, The Use of AI Tools; SRL, Self-Regulated Learning; IB, Innovative Behavior; CT, Critical Thinking; AcInt, Academic Integrity; t, two-tailed t-test statistic; p, significance level.

### 4.5 Summary of standardized path coefficients

**Figure 3:**
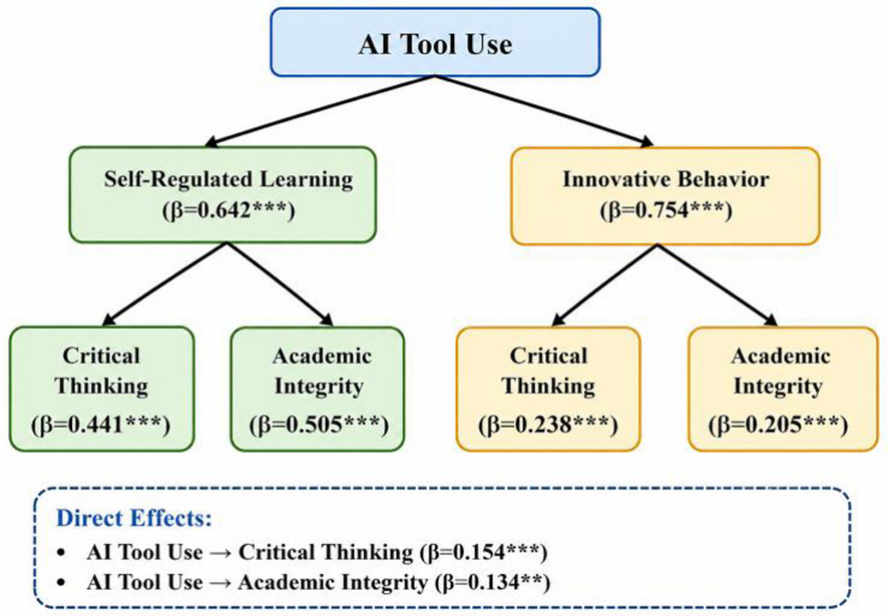
Structural model of AI tool use on learning outcomes. ^***^p < 0.001.

This Figure 3 illustrates the direct and indirect effects of AI tool use on critical thinking and academic integrity, showing stronger indirect effects via self-regulated learning and innovative behavior than direct effects.

## 5 Discussion

This research examined how college students' use of AI tools for academic writing relates to critical thinking and academic integrity. It also tested the parallel indirect pathways through self-regulated learning and innovative behavior within these relationships. The empirical results indicated positive associations between AI tool use and the two academic outcomes and were consistent with the hypothesized indirect associations through self-regulated learning and innovative behavior. From the perspective of SCT, AI tool use can be understood as an environmental condition, self-regulated learning as a form of personal agency, and innovative behavior as active behavioral engagement. The findings suggest that AI tools are not uniformly associated with critical thinking and academic integrity by themselves; rather, the observed associations were stronger in patterns where students regulate their learning, evaluate AI-generated information, and transform AI support into their own writing decisions. These findings help contextualize how AI-related learning behavior links to students' cognitive competence and academic ethics, and extend SCT by showing how environmental affordances, personal regulation, and behavioral engagement are jointly related to academic outcomes in AI-supported writing contexts.

This study found that the use of AI tools in academic writing was positively associated with critical thinking, which is consistent with the findings of Poce (2024) and provides support for H1. However, the evidence is mixed. Essien et al. (2024) found that the positive pattern related to AI text generators and critical thinking was primarily observed at lower cognitive levels of Bloom's taxonomy, with less pronounced improvements at higher-order levels. Systematic review evidence (Zhai et al., 2024) further shows that negative cognitive patterns related to over-reliance on AI tend to co-occur with individual differences such as the level of active engagement. These conditional patterns suggest that the observed relationships may vary with factors such as task complexity and cognitive readiness. AI feedback and revision suggestions often co-occur with students checking coherence and evaluating arguments, which aligns with critical engagement (Essien et al., 2024). From an SCT perspective, this result is consistent with viewing AI tools as environmental resources that co-occur with students' active processing, questioning, and reorganization of information provided by these tools (Bandura, 2001). In addition, AI supported writing workflows may coincide with more occasions for reflection and self-evaluation, as students compare alternatives, revise drafts, and adjust their work against self-set goals (Sardi et al., 2025). AI support may also co-occur with higher motivation and engagement in writing tasks, which may be linked to more sustained attention to analysis and reasoning (Xu and Liu, 2025). Overall, these patterns indicate a co-occurrence between AI tool use, when used as support within students' own decision making, and reflective revision practices that are closely tied to critical thinking. Alternatively, students who possess stronger critical thinking abilities may tend to use AI tools more reflectively, instead of the opposite direction (Shen and Teng, 2024).

Although the use of AI tools was positively associated with academic integrity, the result did not provide empirical support for H2. This finding contrasts with Cotton et al. (2024), yet is consistent with the results reported by Hysaj et al. (2024). A large number of prior studies have emphasized the potential hazards of AI application, holding that excessive dependence on generative AI may induce academic misconduct and erode students' awareness of abiding by academic norms (Eke, 2023). However, the positive association observed in this study should be interpreted cautiously. First, the direct association between AI tool use and academic integrity was statistically significant but relatively small, suggesting that AI use should not be understood as a strong or automatic indicator of academic integrity. Second, this result does not mean that AI tools directly improve students' academic integrity. Rather, it may indicate that students who use AI tools in more regulated and transparent ways also report stronger integrity-related attitudes. AI supported feedback and writing assistance can be integrated into the writing process in ways that coincide with active engagement with content, including checking wording, revising structure, and monitoring adherence to academic standards (Liu et al., 2024). AI tools may also be used alongside routine aspects of writing, which can coincide with less time pressure and more deliberate attention to attribution and revision practices (Rasul et al., 2024). In addition, when students paraphrase, verify, and document AI generated material rather than adopting it uncritically, these practices align with responsible use and may co-occur with lower risks of plagiarism (Karkoulian et al., 2024). Another possible explanation is that students with stronger academic integrity may be more likely to use AI tools as a supplement rather than as a substitute for their own writing. Thus, the positive link may partly reflect students' pre-existing integrity orientations and responsible use habits. Moreover, because academic integrity was measured through self-reported attitudes rather than observed misconduct behaviors, the result may also be influenced by students' tendency to present themselves as norm-compliant. SCT helps explain this finding by emphasizing that ethical behavior emerges from the interaction between environmental affordances, personal self-regulation, and behavioral choices. In this sense, AI tools do not automatically weaken or strengthen academic integrity; their association with integrity depends on how students monitor authorship, apply citation norms, and make responsible writing decisions (Bandura, 2001). Overall, the findings show that AI tool use can co-occur with integrity supportive practices when accompanied by clear norms and self-monitoring.

The results indicate that self-regulated learning shows an indirect association in the relationship between AI tool use and critical thinking, which is consistent with H3. This finding, together with Sardi et al. (2025) and Zhou et al. (2024), shows a pattern: AI tool use and self-regulation are associated, and self-regulation and critical thinking are also associated in this sample. One interpretation: personalized assistance and instant responses from AI tools might appear alongside goal setting, progress tracking, and reflection. These three activities are key parts of self-regulated learning (Zimmerman, 2002). This pathway is central to SCT because it shows how students' personal agency may connect environmental input from AI tools with cognitive engagement in writing (Bandura, 2001). When students review AI suggestions, compare alternatives, and revise their drafts against self-set standards, they engage in iterative checking of arguments, evidence, and coherence. Such monitoring and reflective adjustment are closely aligned with critical thinking in academic writing (Ng et al., 2024). Overall, the results show that AI tool use has a stronger link to critical thinking when combined with students‘ self-regulation and reflective revision habits. Yet, due to the cross-sectional design, we cannot tell if self-regulation comes before AI use or if students who already have stronger self-regulation just tend to use AI tools more effectively.

The analysis also indicates that self-regulated learning shows an indirect association in the relationship between AI tool use and academic integrity, a finding that is consistent with H4. This result aligns with the findings of Sardi et al. (2025) and Simon and Pavlin-Bernardic (2024), suggesting that AI tool use is associated with academic integrity through students' self-regulation in this model. From the perspective of SCT, this finding suggests that self-regulation may represent an intermediary process associated with both AI tool use and norm-aligned writing practices, rather than a causal mechanism established by the present design (Bandura, 2001). When students set boundaries for AI assistance, monitor attribution and originality, and reflect on whether their work meets academic standards, these practices align with higher integrity in written assignments (Sarbini et al., 2023). In AI supported writing contexts, such monitoring and reflection can involve tracking how AI outputs are used, revising or paraphrasing content, and documenting sources and assistance. Overall, the findings show that AI tool use is more consistently associated with academic integrity when it co-occurs with students' goal setting, self-monitoring, and reflective judgement during writing.

This study highlights that innovative behavior shows an indirect association between the use of AI tools and critical thinking, providing support for H5. This finding is consistent with the research of Ma et al. (2024) and Li et al. (2024). One interpretation is that AI tools can be used for idea exploration and alternative framing, which may be associated with students moving beyond conventional writing routines and developing more original arguments. Such innovation oriented engagement is compatible with critical thinking, as it involves comparing options, justifying choices, and restructuring content during writing (Fassbender, 2024). In SCT terms, innovative behavior represents the behavioral engagement component of the model, showing how students actively transform environmental input rather than passively receiving it (Bandura, 2001). In addition, when students evaluate AI generated suggestions rather than accepting them at face value, they engage in analysis and judgement that aligns with critical thinking in academic work (Hendekci, 2025). Overall, the findings show that AI tool use is more closely associated with critical thinking when it co-occurs with innovation-oriented engagement during writing. It is also possible that students with higher innovative behavior are more likely to explore AI tools in depth, rather than AI use being associated with innovation.

Additionally, the results indicate that innovative behavior shows an indirect association in the relationship between AI tool use and academic integrity, which is consistent with H6. This result aligns with the findings of Ma et al. (2024) and Su and He (2023). From an SCT perspective, this finding further indicates that behavioral engagement is not only linked to cognitive outcomes but also to ethical orientations in AI-supported writing (Bandura, 2001). In AI supported writing, students may use tools to explore alternatives, integrate sources, and articulate novel viewpoints, which may be associated with originality in both ideas and expression. Such originality oriented engagement has been linked to stronger integrity related practices, including reduced reliance on unacknowledged text and greater attention to attribution (Eshet and Margaliot, 2022). It may also coincide with closer alignment to academic norms, as students who invest in developing their own contributions often monitor standards more carefully (Krou et al., 2021). Overall, the findings indicate that AI tool use is positively associated with academic integrity when it co-occurs with innovation-oriented engagement during writing. It is also possible that students with higher innovative behavior are more likely to explore AI tools in depth, rather than AI use causing innovation.

Taken together, these findings are consistent with the application of SCT to AI-supported writing as a dynamic relation among environmental conditions, personal regulation, and behavioral engagement. The associations between AI tool use and academic outcomes were not limited to direct links. Instead, self-regulated learning and innovative behavior were associated with patterns in which students may work with AI tools in ways that are connected with reflective reasoning and responsible academic practice.

## 6 Implications

### 6.1 Theoretical implications

This study extends the application of SCT to AI supported academic writing by examining how environmental conditions, personal regulation, and behavioral engagement are jointly associated with students' academic competencies. AI tool use is specified as a feature of the writing environment, while self-regulated learning and innovative behavior are positioned as intermediate processes linked to critical thinking and academic integrity. This framing offers a coherent way to interpret AI supported writing beyond a narrow focus on writing performance.

This work also advances educational technology research by placing critical thinking and academic integrity together in one pathway model. Prior studies have often examined these outcomes separately or concentrated on direct associations between technology use and performance indicators. By identifying common indirect association pathways, this framework offers one way to interpret how cognitive and ethical aspects of academic ability might appear together with students' self-regulation and innovation-focused involvement when writing with AI support.

### 6.2 Practical implications

At the institutional and course level, universities may make AI supported writing expectations more usable by attaching brief, assignment specific guidance that distinguishes permitted support, support requiring disclosure, and non-permitted uses. A short disclosure Appendix can be required when AI is used, noting the tool, purpose, and how outputs were revised, which may be useful for reducing ambiguity around authorship and attribution. Colleges and universities should formulate standardized AI application guidelines, guide students to integrate AI resources into learning rationally, and build sound management and supervision mechanisms to support responsible AI use while maintaining campus academic integrity norms.

In teaching and assessment, AI use can be embedded in routine writing pedagogy through simple checkpoints that foreground planning, monitoring, and reflection, such as a goal and outline before drafting, a short revision note explaining how AI suggestions were handled, and a final statement on source use and acknowledgment. Rubrics can include a small number of observable criteria, for example revision quality, source verification, and transparency of tool use, rather than relying on AI detection. Finally, integrity education can be updated with a short module covering disclosure rules, paraphrasing, and source checking in AI supported writing, which may help students apply these norms more consistently. Teachers may develop specific classroom tasks that help students use AI tools wisely for idea development and content improvement. Such tasks may also create more opportunities for students to exercise their own judgment and analysis skills, thereby supporting the growth of critical thinking in real academic work.

## 7 Limitations and future research directions

While this research provides significant contributions to understanding the associations between AI tools, critical thinking, and academic integrity, it is important to acknowledge certain limitations. To begin with, the reliance on self-administered questionnaires may introduce potential biases such as subjectivity and social desirability. This issue is particularly relevant to academic integrity, because students may report more norm-compliant attitudes or responsible AI-use practices than they actually hold or perform. Although the survey emphasized anonymity and voluntary participation, these procedures cannot fully remove the possibility of socially desirable responses. In addition, Harman's single-factor test only addresses common method bias and does not directly test social desirability bias. Future studies should include social desirability scales, behavioral indicators of academic integrity, or qualitative methods such as interviews to provide a more comprehensive assessment. Second, relying on cross-sectional data restricts the potential to explore directional or causal patterns or track developmental changes across time. Although this study found significant links between the variables, we cannot tell if AI use comes before the measured outcomes, or if students who already have stronger critical thinking, self-regulation, and academic honesty tend to use AI tools more effectively. Longitudinal studies should track students over time to see how ongoing AI use relates to changes in critical thinking and academic honesty. Third, the study focused exclusively on college students from Chinese higher education. Students' use of AI tools and their understanding of academic integrity may be influenced by local academic norms, writing assessment practices, and institutional expectations regarding AI-assisted writing. Therefore, the findings may not be directly generalizable to learners from different educational stages, cultural backgrounds, or AI governance contexts. Future studies are encouraged to include samples from varied educational stages, regions, and countries to further examine the applicability of these relationships across contexts. Fourth, the sample was drawn from five universities and multiple classes. Students within the same university or class may share similar institutional policies, instructional environments, or peer influences, which can introduce clustering effects. The PLS-SEM analysis did not explicitly model such clustering, which may affect the precision of standard error estimates. Future research should consider multilevel modeling or cluster-robust variance estimation to address the nested structure of educational data. Fifth, although Q^2^ values confirmed predictive relevance, the relatively high Q^2^ for innovative behavior (0.567) should be interpreted with caution. Multicollinearity checks (VIF < 5) and sample size (*N* = 946) ruled out overfitting as a major concern. Nevertheless, future research could apply cross-validation or test the model on independent samples to further verify its predictive validity and generalizability. Sixth, several scales were adapted to AI-specific writing contexts. All adapted items explicitly referred to AI tool use (e.g., “During the use of AI tools...”), supporting content validity. However, we did not include measures of general academic tendencies (e.g., general critical thinking) to test discriminant validity. Thus, the observed associations may partly reflect students' pre-existing general abilities rather than AI-specific practices. Future research should include both general and AI-specific measures to address this limitation. Seventh, this study treated AI tools as a single construct. Although we provided an operational definition of AI tools, the measure did not distinguish between different types of tools, such as generative AI for text production, grammar-checking tools, and literature-mapping tools. These tools may support academic writing in different ways and may have different associations with critical thinking and academic integrity. Therefore, the findings should be interpreted as reflecting students' overall AI tool use rather than the specific associations linked to particular AI applications. Future research should examine different types of AI tools separately to provide a more fine-grained understanding of their academic implications.

## 8 Conclusion

Grounded in SCT, this study examined how AI tool use in academic writing is associated with critical thinking and academic integrity alongside self-regulated learning and innovative behavior. The findings indicate that AI tool use was positively associated with critical thinking and academic integrity, alongside stronger self-regulation and innovation-oriented engagement. These results offer further insight into the correlational patterns underlying the psychological and behavioral aspects of AI-assisted academic writing. They also point to practical ideas for using AI tools in educational settings alongside cognitive and ethical skills. Nevertheless, limitations related to the use of a single-group sample and a cross-sectional design limit the generalizability and do not allow directional or causal interpretations of the observed associations. It remains possible that students with stronger self-regulated learning, critical thinking, and academic integrity are more likely to use AI tools strategically, rather than AI use directly influencing these competencies. Future research is encouraged to use more diverse samples, adopt longitudinal designs, and examine additional contextual and individual factors related to AI tool use in educational settings.

## Data Availability

The raw data supporting the conclusions of this article will be made available by the authors, without undue reservation.

## References

[B1] Akcaoglu M. Ö. Mor E. Külekçi E. (2023). The mediating role of metacognitive awareness in the relationship between critical thinking and self-regulation. Think. Skills Creat. 47:101187. doi: 10.1016/j.tsc.2022.101187

[B2] Bandura A. (2001). Social cognitive theory: an agentic perspective. Annu. Rev. Psychol. 52, 1–26. doi: 10.1146/annurev.psych.52.1.1 11148297

[B3] Bearman M. Ajjawi R. (2023). Learning to work with the black box: Pedagogy for a world with artificial intelligence. Br. J. Educ. Technol. 54, 1029–1045. doi: 10.1111/bjet.13337

[B4] Bin-Nashwan S. A. Sadallah M. Bouteraa M. (2023). Use of ChatGPT in academia: Academic integrity hangs in the balance. Technol. Soc. 75:102370. doi: 10.1016/j.techsoc.2023.102370

[B5] Bittle K. El-Gayar O. (2025). Generative AI and academic integrity in higher education: a systematic review and research agenda. Information 16:296. doi: 10.3390/info16040296

[B6] Cotton D. R. Cotton P. A. Shipway J. R. (2024). Chatting and cheating: ensuring academic integrity in the era of ChatGPT. Innov. Educ. Teach. Int. 61, 189–203. doi: 10.1080/14703297.2023.2190148

[B7] Currie G. M. (2023). Academic integrity and artificial intelligence: is ChatGPT hype, hero or heresy? Seminars in nuclear medicine. Advance online publication. doi: 10.1053/j.semnuclmed.2023.04.008 37225599

[B8] Dahri N. A. Yahaya N. Al-Rahmi W. M. Aldraiweesh A. Alturki U. Almutairy S. . (2024). Extended TAM based acceptance of AI-Powered ChatGPT for supporting metacognitive self-regulated learning in education: a mixed-methods study. Heliyon 10:e29317. doi: 10.1016/j.heliyon.2024.e29317 38628736 PMC11016976

[B9] Eke D. O. (2023). ChatGPT and the rise of generative AI: threat to academic integrity? J. Resp. Technol. 13:100060. doi: 10.1016/j.jrt.2023.100060

[B10] El-Sayed A. A. I. Alsenany S. A. Almalki R. S. E. Asal M. G. R. (2025). Fostering creativity-nurturing behaviors among nurse educators: investigating the interplay between evidence-based practice climate and artificial intelligence competence self-efficacy. Nurse Educ. Today 151:106734. doi: 10.1016/j.nedt.2025.106734 40215710

[B11] Eshet Y. Margaliot A. (2022). Does creative thinking contribute to the academic integrity of education students? Front. Psychol. 13:925195. doi: 10.3389/fpsyg.2022.925195 35992454 PMC9386246

[B12] Essien A. Bukoye O. T. O'Dea C. Kremantzis M. (2024). The influence of AI text generators on critical thinking skills in UK business schools. Stud. High. Educ. 49, 865–882. doi: 10.1080/03075079.2024.2316881

[B13] Fassbender W. J. (2024). I can almost recognize its voice: AI and its impact on ethical teacher-centaur labor. Eng. Teach. Practice Critique 23, 1–15. doi: 10.1108/ETPC-08-2023-0101

[B14] Fornell C. Larcker D. F. (1981). Evaluating structural equation models with unobservable variables and measurement error. J. Market. Res. 18, 39–50. doi: 10.1177/002224378101800104

[B15] Gefen D. Rigdon E. E. Straub D. (2011). Editor's comments: an update and extension to SEM guidelines for administrative and social science research. MIS Q. 35, iii-xiv. doi: 10.2307/23044042

[B16] Gonsalves C. (2024). Generative AI's impact on critical thinking: revisiting bloom's taxonomy. J. Market. Educ. 46, 115–127. doi: 10.1177/02734753241305980

[B17] Hair Jr J. Hair Jr J. F. Hult G. T. M. Ringle C. M. Sarstedt M. (2021). A Primer on Partial Least Squares Structural Equation Modeling (PLS-SEM). Thousand Oaks, CA: Sage publications. doi: 10.1007/978-3-030-80519-7

[B18] Hair J. F. Ringle C. M. Sarstedt M. (2011). PLS-SEM: Indeed a silver bullet. J. Market. Theor. Pract. 19, 139–152. doi: 10.2753/MTP1069-6679190202

[B19] Helzer E. G. Cohen T. R. Kim Y. Iorio A. Aven B. (2024). Moral beacons: understanding moral character and moral influence. J. Pers. 92, 735–752. doi: 10.1111/jopy.12865 37548060

[B20] Hendekci A. (2025). Innovation, curiosity, exploration, and critical thinking dispositions of nursing students who have and have not taken an innovative thinking course. Nurs. Health Sci. 27, 1–10. doi: 10.1111/nhs.70127 PMC12040766 40301123

[B21] Hooda M. Rana C. Dahiya O. Rizwan A. Hossain M. S. (2022). Artificial intelligence for assessment and feedback to enhance student success in higher education. Math. Problems Eng. 2022:5215722. doi: 10.1155/2022/5215722

[B22] Hosseini M. Gao C. A. Liebovitz D. M. Carvalho A. M. Ahmad F. S. Luo Y. . (2023). An exploratory survey about using ChatGPT in education, healthcare, and research. PLoS ONE 18:e0292216. doi: 10.1371/journal.pone.0292216 37796786 PMC10553335

[B23] Hysaj A. Freeman M. Hamam D. (2024). “Using AI tools to enhance academic writing and maintain academic integrity,” in Proceedings of the 16th International Conference on Social Computing and Social Media (SCSM). Berlin: Springer. doi: 10.1007/978-3-031-61305-0_4

[B24] Jankovic A. Kulic D. (2025). Use and misuse of chatgpt in academic writing among the english language students. Inform. Technol. Learn. Tools 105, 1–12. doi: 10.33407/itlt.v105i1.5955

[B25] Ji Y. Zhong M. X. Lyu S. Li T. T. Niu S. J. Zhan Z. H. (2025). How does AI literacy affect individual innovative behavior: the mediating role of psychological need satisfaction, creative self-efficacy, and self-regulated learning. Educ. Inform. Technol. Advance online publication. doi: 10.1007/s10639-025-13437-4

[B26] Jin F. Z. Lin C. H. Lai C. (2025). Modeling AI-assisted writing: how self-regulated learning influences writing outcomes. Comput. Human Behav. 165:108538. doi: 10.1016/j.chb.2024.108538

[B27] Karkoulian S. Sayegh N. Sayegh N. (2024). ChatGPT unveiled: understanding perceptions of academic integrity in higher education - a qualitative approach. J. Acad. Ethics. Advance online publication. doi: 10.1007/s10805-024-09543-6

[B28] Kline R. B. (2018). Response to Leslie Hayduk's review of principles and practice of structural equation modeling. Can. Stud. Popul. 45, 188–195. doi: 10.25336/csp29418

[B29] Kline R. B. (2023). Principles and Practice of Structural Equation Modeling. New York, NY: Guilford Press.

[B30] Kong S. C. Lee J. C. K. Tsang O. (2024). A pedagogical design for self-regulated learning in academic writing using text-based generative artificial intelligence tools: 6-P pedagogy of plan, prompt, preview, produce, peer-review, portfolio-tracking. Res. Pract. Technol. Enhanced Learn. 19, 1–20. doi: 10.58459/rptel.2024.19030

[B31] Krou M. R. Fong C. J. Hoff M. A. (2021). Achievement motivation and academic dishonesty: a meta-analytic investigation. Educ. Psychol. Rev. 33, 427–461. doi: 10.1007/s10648-020-09557-7

[B32] Leguina A. (2015). A primer on partial least squares structural equation modeling (PLS-SEM). Int. J. Res. Method Educ. 38, 220–221. doi: 10.1080/1743727X.2015.1005806

[B33] Li K. Wijaya T. T. Chen X. Y. Harahap M. S. (2024). Exploring the factors affecting elementary mathematics teachers' innovative behavior: an integration of social cognitive theory. Sci. Rep. 14:52604. doi: 10.1038/s41598-024-52604-4 PMC10808225 38267501

[B34] Liu R. Zenke C. Liu C. Holmes A. Thornton P. Malan D. J. (2024). “Teaching CS50 with AI: leveraging generative artificial intelligence in computer science education,” in Proceedings of the 55th ACM Technical Symposium on Computer Science Education. doi: 10.1145/3626252.3630938

[B35] Lodge J. M. de Barba P. Broadbent J. (2023). Learning with generative artifiicial intelligence within a network of co- regulation. J. Univ. Teach. Learn. Practice 20, 1–15. doi: 10.53761/1.20.7.02

[B36] Lund B. D. Lee T. H. Mannuru N. R. Arutla N. (2025). AI and academic integrity: exploring student perceptions and implications for higher education. J. Acad. Ethics. 23, 1545-1565. doi: 10.1007/s10805-025-09613-3

[B37] Ma K. Zhang Y. Hui B. H. (2024). How does AI affect college? the impact of AI usage in college teaching on students' innovative behavior and well-being. Behav. Sci. 14:1223. doi: 10.3390/bs14121223 39767364 PMC11673235

[B38] Macfarlane B. Zhang J. Pun A. (2014). Academic integrity: a review of the literature. Stud. High. Educ. 39, 339–358. doi: 10.1080/03075079.2012.709495

[B39] Man Tang P. Koopman J. McClean S. T. Zhang J. H. Li C. H. De Cremer D. . (2022). When conscientious employees meet intelligent machines: an integrative approach inspired by complementarity theory and role theory. Acad. Manag. J. 65, 1603–1632. doi: 10.5465/amj.2020.1516

[B40] McAlister A. R. Alhabash S. Yang J. (2024). Artificial intelligence and ChatGPT: exploring current and potential future roles in marketing education. J. Market. Commun. 30, 1–18. doi: 10.1080/13527266.2023.2289034

[B41] Ng D. T. K. Tan C. W. Leung J. K. L. (2024). Empowering student self-regulated learning and science education through ChatGPT: a pioneering pilot study. Br. J. Educ. Technol. 55, 1–15. doi: 10.1111/bjet.13454

[B42] Ng T. W. Lucianetti L. (2016). Within-individual increases in innovative behavior and creative, persuasion, and change self-efficacy over time: a social–cognitive theory perspective. J. Appl. Psychol. 101, 14–34. doi: 10.1037/apl0000029 26052714

[B43] Pintrich P. R. (1991). A manual for the use of the motivated strategies for learning questionnaire (MSLQ). doi: 10.1037/t09161-000

[B44] Pintrich P. R. (2000). Multiple goals, multiple pathways: the role of goal orientation in learning and achievement. J. Educ. Psychol. 92, 544–555. doi: 10.1037/0022-0663.92.3.544

[B45] Poce A. (2024). “Using AI for critical thinking assessment: a digital humanities education experience,” in 17th Learning Ideas Conference, eds. D. Guralnick, M. E. Auer, and A. Poce (Springer). doi: 10.1007/978-3-031-73427-4_9

[B46] Podsakoff P. M. MacKenzie S. B. Lee J. Y. Podsakoff N. P. (2003). Common method biases in behavioral research: a critical review of the literature and recommended remedies. J. Appl. Psychol. 88, 879–903. doi: 10.1037/0021-9010.88.5.879 14516251

[B47] Qadir J. (2023). “Engineering education in the era of ChatGPT: Promise and pitfalls of generative AI for education,” in 2023 IEEE Global Engineering Education Conference (EDUCON) (IEEE). doi: 10.1109/EDUCON54358.2023.10125121

[B48] Ramdani Z. (2018). Construction of academic integrity scale. Int. J. Res. Stud. Psychol. 7, 1–10. doi: 10.5861/ijrsp.2018.3003

[B49] Rasul T. Nair S. Kalendra D. Balaji M. S. Santini F. D. Ladeira W. Jr. . (2024). Enhancing academic integrity among students in GenAI Era:A holistic framework. Int. J. Manag. Educ. 22:101041. doi: 10.1016/j.ijme.2024.101041

[B50] Ruiz-Rojas L. I. Salvador-Ullauri L. Acosta-Vargas P. (2024). Collaborative working and critical thinking: adoption of generative artificial intelligence tools in higher education. Sustainability 16:5367. doi: 10.3390/su16135367

[B51] Sarbini S. Supriyatin T. Sukaesih E. Kusnawan A. Yunus A. R. B. (2023). Religiosity of mediators between self regulated learning and academic integrity. Psikis: J. Psikologi Islami 9, 40–51. doi: 10.19109/psikis.v9i1.14981

[B52] Sardi J. Darmansyah C.andra, O. Yuliana D. F. Habibullah Yanto, D. T. P. Eliza F. (2025). How generative AI influences students' self-regulated learning and critical thinking skills? a systematic review. Int. J. Eng. Pedagog. 15, 94–108. doi: 10.3991/ijep.v15i1.53379

[B53] Sarstedt M. Ringle C. M. Hair J. F. (2021). “Partial least squares structural equation modeling,” in Handbook of Market Research (Berlin: Springer), 587-632. doi: 10.1007/978-3-319-05542-8_15-2

[B54] Scriven M. Paul R. (1987). “Critical thinking,” in The 8th Annual International Conference on Critical Thinking and Education Reform, CA.

[B55] Shen X. Teng M. F. (2024). Three-wave cross-lagged model on the correlations between critical thinking skills, self-directed learning competency and AI-assisted writing. Think. Skills Creat. 52:101524. doi: 10.1016/j.tsc.2024.101524

[B56] Simon J. Pavlin-Bernardic N. (2024). Academic dishonesty in written assignments - the role of contextual and motivational factors. Metodički Ogledi 31, 1–18. doi: 10.21464/mo.31.1.12

[B57] Su P. He M. (2023). The impact of innovative behaviors on academic misconduct among graduate students: a mediated moderation model. Front. Psychol. 14:1276700. doi: 10.3389/fpsyg.2023.1276700 37901074 PMC10600469

[B58] Sudibjo N. Prameswari R. K. (2021). The effects of knowledge sharing and person–organization fit on the relationship between transformational leadership on innovative work behavior. Heliyon 7:e07338. doi: 10.1016/j.heliyon.2021.e07334 34195436 PMC8239725

[B59] Van Slyke C. Johnson R. D. Sarabadani J. (2023). Generative artificial intelligence in information systems education: Challenges, consequences, and responses. Commun. Assoc. Inform. Syst. 53, 1–17. doi: 10.17705/1CAIS.05301

[B60] Wang D. Xing X. H. Wu X. B. (2012). The healthy lifestyle scale for university students: development and psychometric testing. Austr. J. Primary Health 18, 339–345. doi: 10.1071/PY11107 22950906

[B61] Xu J. Liu Q. W. (2025). Uncurtaining windows of motivation, enjoyment, critical thinking, and autonomy in AI-integrated education: Duolingo Vs. ChatGPT. Learn. Motiv. 89:102100. doi: 10.1016/j.lmot.2025.102100

[B62] Zhai C. P. Wibowo S. Li L. D. (2024). The effects of over-reliance on AI dialogue systems on students' cognitive abilities: a systematic review. Smart Learn. Environ. 11:16. doi: 10.1186/s40561-024-00316-7

[B63] Zhang Q. C. Liao G. L. Ran X. Y. Wang F. W. (2025). The impact of AI usage on innovation behavior at work: the moderating role of openness and job complexity. Behav. Sci. 15:491. doi: 10.3390/bs15040491 40282112 PMC12024388

[B64] Zhao M. Maat S. M. Azman N. Zheng E. (2024). The relationship between faculty support, academic self-efficacy, academic emotions, and online learning performance among university students in China. Sage Open 14, 1–12. doi: 10.1177/21582440241304928

[B65] Zhou X. Teng D. Al-Samarraie H. (2024). The mediating role of generative AI self-regulation on students' critical thinking and problem-solving. Educ. Sci. 14:1213. doi: 10.3390/educsci14121302

[B66] Zimmerman B. J. (2000). “Attaining self-regulation: a social cognitive perspective,” in Handbook of Self-Regulation, eds. M. Boekaerts, P. R. Pintrich, and M. Zeidner (San Diego, CA: Academic Press), 3–39. doi: 10.1016/B978-012109890-2/50031-7

[B67] Zimmerman B. J. (2002). Becoming a self-regulated learner: an overview. Theor. Pract. 41, 64–70. doi: 10.1207/s15430421tip4102_2

