# Supplementary material for: The use of AI tools in academic writing and its associations with college students' critical thinking and academic integrity: a path analysis involving self-regulated learning and innovative behavior
Source: Front Psychol. 2026 Jul 8;17:1845571. doi: 10.3389/fpsyg.2026.1845571 (PMC13390628; doi:10.3389/fpsyg.2026.1845571)
Supplement: Supplementary file 1 [file Supplementary_file_1.docx]

**Appendix A: Measurement Scales**

| Construct | Item | |
| --- | --- | --- |
| The Use of AI Tools | I spent most of my academic writing time working with artificial intelligence. |  |
|  | I used artificial intelligence to carry out most of my academic writing tasks. |  |
|  | I worked with artificial intelligence in making major academic writing decisions. |  |
| Self-Regulated Learning | When I use GenAI to read for this course, I make up questions to help focus my reading. |  |
|  | If the materials provided by GenAI are difficult to understand, I am able to change the way I read the material. |  |
|  | I try to change the way I use GenAI for study in order to fit the course requirements and instructors' teaching style. |  |
|  | When I use GenAI, I try to think through a topic and decide what I am supposed to learn from it rather than just reading it over. |  |
| Innovative Behavior | I create new ideas for improvements. |  |
|  | I search out new working methods, techniques, or instruments. |  |
|  | I generate original solutions to problems. |  |
|  | I mobilize support for innovative ideas. |  |
|  | I acquire approval for innovative ideas. |  |
|  | I make important organizational members enthusiastic for innovative ideas. |  |
|  | I transform innovative ideas into useful applications. |  |
|  | I introduce innovative ideas into the work environment in a systematic way. |  |
|  | I evaluate the utility of innovative ideas. |  |
| Critical Thinking | I often find myself questioning things I read from GenAI to decide if I find them convincing. |  |
|  | When a theory, interpretation or conclusion is presented in GenAI, I try to decide if there is good supporting evidence. |  |
|  | I treat GenAI content as a starting point and try to develop my own ideas about it. |  |
|  | I try to play around with ideas of my own related to what I am learning in GenAI. |  |
|  | Whenever I read an assertion or conclusion generated by GenAI, I think about possible alternatives. |  |
| Academic Integrity | For me to be honest it starts from myself. |  |
|  | Honesty trains us to believe in our abilities. |  |
|  | I really appreciate friends who do the tasks with their own ability. |  |
|  | I am sure that any work done honestly results will be satisfactory. |  |
|  | Originality of ideas is an important thing to have when writing. |  |
|  | I am happy to pass the course material to my friend. |  |
|  | I am glad when a friend asks my idea in doing the lecture work. |  |
|  | I am active to participate in academic activities inside and outside of the campus. |  |
|  | I love studying other people's research results. |  |
|  | All students have equal opportunities to get involved in campus activities. |  |
|  | Regular academic evaluation is very important in the learning process. |  |
|  | Trusting each other's friends is a solid foundation for collaboration on campus. |  |
|  | I like to discuss how to cite the reference sources that lecturers present in the classroom. |  |
|  | For me preparing the material before the lecture is a natural thing. |  |
|  | I enjoy discussing college assignments with friends. |  |
|  | Getting a scholarship is like having a responsibility to serve the nation. |  |
|  | I feel a good image of campus is a shared responsibility. |  |
